# A trick for a treat: False smut pathogen manipulates plant defense to gain access to rice flower

**DOI:** 10.1093/plcell/koae048

**Published:** 2024-02-15

**Authors:** Kutubuddin A Molla

**Affiliations:** Assistant Features Editor, The Plant Cell, American Society of Plant Biologists; ICAR-National Rice Research Institute, Cuttack, India

Once a minor disease, rice false smut, caused by *Ustilaginoidea virens*, has recently become one of the most important diseases in rice-growing areas of the world. In India, yield loss due to false smut reaches up to 49%, while in China, the average yield loss accounts for 158.6 million kg/year (Sun et al. 2020). In addition to affecting food security, the false smut pathogen threatens food safety by producing mycotoxins such as ustiloxins and ustilaginoidins (Zhou et al. 2012). Control of rice false smut primarily relies on the application of fungicides. *U. virens* is a flower-infecting fungus that infects stamen and pistil by invading via the gap between lemma and palea. However, how rice flowers defend and how the pathogen overcomes plant defenses are largely unknown.

In this issue, **Guo-Bang Li and colleagues** (Li et al. 2024) delineated how *U. virens* suppresses rice chitin-triggered immunity and identified a core virulence factor, UvGH18.1. The authors’ previous work identified a *U. virens*-secreted cytoplasmic effector, UvCBP1, that facilitates the infection of rice flowers (Li et al. 2022). UvCBP1 was predicted to contain a glycoside hydrolase family 18 (GH18) domain and was consequently renamed UvGH18.1. To investigate the function of UvGH18.1, the authors generated knockout mutants. The *uvgh18.1* mutant failed to develop false smut balls, the typical symptom of the disease, on rice panicles (see Fig.). Upon complementation with a functional copy of UvGH18.1, the mutant's pathogenicity was restored. Polymorphism analysis of 50 field isolates from China, Japan, and Nepal showed that the UvGH18.1 is highly conserved in natural populations.

**Figure.**
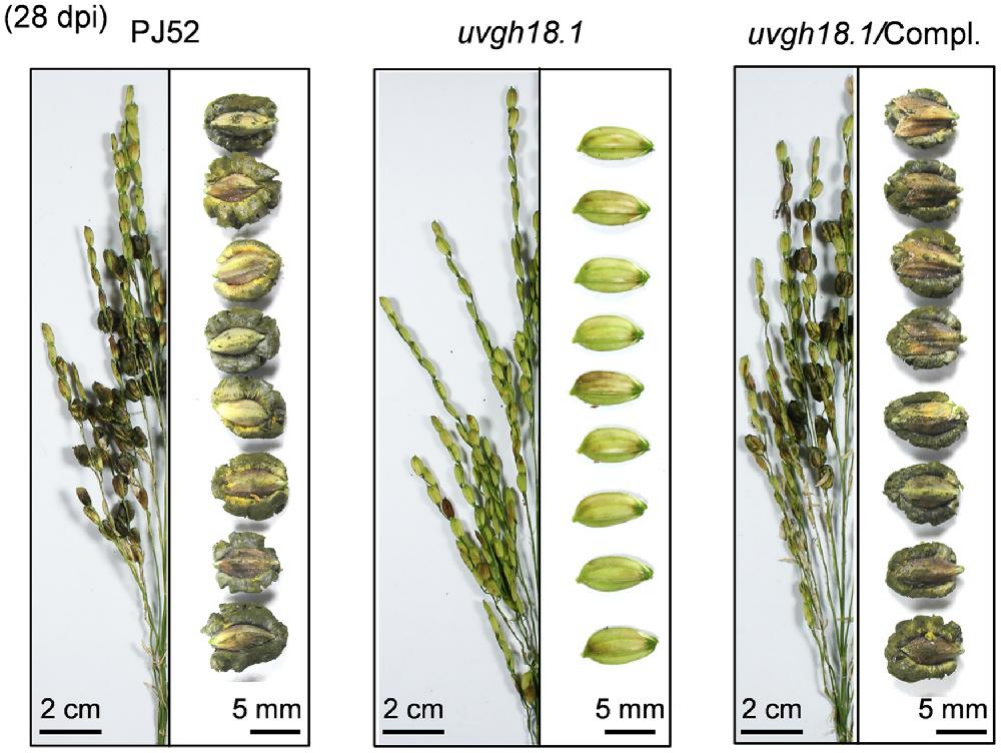
A disruption of UvGH18.1 function abolishes the ability of false smut ball formation by *U. virens* in rice panicles. Complemented strain restores the ability for false smut ball formation. Adapted from Li et al. (2024), Figure 1D.

Next, the authors demonstrated that UvGH18.1 acts as a chitinase, degrading immunogenic chitin oligomers (with 6 monomers) into smaller, nonimmunogenic molecules (with ≤4 monomers). Plants typically generate a burst of reactive oxygen species (ROS) upon sensing immunogenic chitin. Interestingly, chitin treated with recombinant UvGH18.1 produced significantly less ROS in *Nicotiana benthamiana* compared to chitin treated with buffer. In contrast, recombinant UvGH18.1^mcc^, which lacks chitinase and chitin-binding activity, lost the ability to inhibit chitin from generating ROS. However, UvGH18.1 did not stop the production of ROS triggered by flagellin, showing that UvGH18.1 specifically blocks chitin-triggered immunity.

Cell surface receptors OsCEBiP and OsCERK1 play a crucial role in the antifungal defense of rice. Their dimerization, a crucial step for intracellular immune signaling, is triggered by chitin binding. The authors demonstrated that UvGH18.1 physically interacts with OsCEBiP and OsCERK1. The interaction inhibited the chitin-induced dimerization of OsCEBiP and OsCERK1.

Next, the authors created CRISPR mutants of *OsCEBiP* and *OsCERK1* to investigate their roles in rice defense against *U. virens*. *U. virens* produced more false smut balls on panicles of *oscebip oscerk1* mutants than on wild-type plants, indicating that OsCEBiP and OsCERK1 are necessary for floral resistance to the pathogen. Interestingly, *U. virens* with disrupted UvGH18.1 failed to produce false smut balls in both wild-type and *oscebip oscerk1* mutants.

Through genetic complementation tests, the authors demonstrated that targeting both chitin and chitin receptors, OsCEBiP and OsCERK1, is essential for UvGH18.1 to suppress rice immunity and increase virulence. When wild-type plants were pretreated with chitin, they showed a nearly 50% reduction in fungal growth. However, this was not observed in *oscebip oscerk1* mutant plants. By analyzing H_2_O_2_ accumulation and defense gene expression, the authors further showed that the inducible defense response mainly occurs in lemma and palea, pinpointing the battlefield against the pathogen. In contrast to the optimal defense theory's prediction of constitutive defense in flowers (Zangerl and Rutledge 1996), this study showed that inducible pathogen-triggered immunity occurs in floral organs.

The study by Li et al. (2024) identifies a core effector, UvGH18.1, essential for the pathogenesis of *U. virens*, providing a significant mechanistic understanding of the disease. This valuable information is poised to drive the development of new strategies to combat this emerging threat to rice cultivation. Looking ahead, future research may focus on identifying chemicals capable of inhibiting the function of UvGH18.1, paving the way in managing false smut disease.

## Dive Curated Terms

The following phenotypic, genotypic, and functional terms are of significance to the work described in this paper:

OsCEBiP Gramene: Os03g0133400
OsCEBiP Araport: Os03g0133400

OsCERK1 Gramene: Os08g0538300
OsCERK1 Araport: Os08g0538300
